# 'It just wasn’t going to be heard’: A mixed methods study to compare different ways of involving people with diabetes and health‐care professionals in health intervention research

**DOI:** 10.1111/hex.13061

**Published:** 2020-05-01

**Authors:** Emmy Racine, Fiona Riordan, Eunice Phillip, Grainne Flynn, Sheena McHugh, Patricia M. Kearney

**Affiliations:** ^1^ School of Public Health University College Cork Cork Ireland; ^2^ Independent Patient and Public Involvement Partner Co. Clare Ireland

**Keywords:** consensus process, intervention development, patient and public involvement, user involvement

## Abstract

**Background:**

Guidelines recommend involving intervention users in the intervention development process. However, there is limited guidance on how to involve users in a meaningful and effective way.

**Objective:**

The aim of this Study within a trial was to compare participants’ experiences of taking part in one of three types of consensus meetings—people with diabetes‐only, combined people with diabetes and health‐care professionals (HCPs) or HCP‐only meeting.

**Design:**

The study used a mixed methods convergent design. Quantitative (questionnaire) and qualitative (observation notes and semi‐structured telephone interviews) data were collected to explore participants’ experiences. A triangulation protocol was used to compare quantitative and qualitative findings.

**Participants:**

People with diabetes (recruited via multiple strategies) were randomly assigned to attend the people with diabetes or combined meeting. HCPs (recruited through professional networks) attended the HCP or combined meeting based on their availability.

**Results:**

Sixteen people with diabetes and 15 HCPs attended meetings, of whom 18 participated in a telephone interview. Participants’ questionnaire responses suggested similar positive experiences across the three meetings. Observation and semi‐structured interviews highlighted differences experienced by participants in the combined meeting relating to: perceived lack of common ground; feeling empowered versus undervalued; needing to feel safe and going off task to fill the void.

**Conclusions:**

The qualitative theme ‘needing to feel safe’ may explain the dissonance (disagreement) between quantitative and qualitative data. In this study, involving patients and HCPs simultaneously in a consensus process was not found to be as suitable as involving each stakeholder group separately.

## 1 INTRODUCTION

For interventions to be successfully implemented in practice, they need to be acceptable, engaging and feasible to implement.^1^ Intervention development guidelines recommend involving all appropriate intervention users to maximize the chances of successful implementation.^2^ User involvement is a broad term that includes (but is not limited to) those receiving, eg patients and members of the public and delivering the intervention, eg healthcare professionals (HCPs).

Consensus methods are a way of involving multiple users simultaneously in the intervention development process.^3^, ^4^, ^5^ Different users may have different priorities and preferences when making decisions about the content and delivery of an intervention.^6^, ^7^ For example, patients and members of the public may be concerned about how an intervention will be received by the target population, whereas HCPs may be more concerned about the cost involved (both time and money).^7^ Group dynamics are complex, and some user groups may find it more difficult to voice their priorities and perspectives compared with others.^8^ Despite increasing emphasis on user involvement, limited guidance exists on how to involve users in a meaningful and effective way. To our knowledge, no research has been conducted on patients and HCPs experiences of being involved in consensus methods and whether their experiences differ according to group composition.

The aim of this Study Within A Trial was to compare participants’ experiences of taking part in one of three types of consensus meetings—people with diabetes‐only, combined people with diabetes and HCPs or HCP‐only meeting.

### 1.1 METHODS

This Study Within A Trial (SWAT) was conducted within the on‐going Improving Diabetes Eye‐screening Attendance (IDEAs) study. IDEAs is a feasibility study of a multifaceted intervention in general practice targeting HCPs and people with diabetes to improve the uptake of retinopathy screening. As part of the development phase of IDEAs, three separate consensus meetings were held to discuss the acceptability and feasibility of the proposed intervention content and suitable modes of delivery. Recommendations from each meeting were used to refine intervention components that could be delivered in general practice. The first consensus meeting consisted of people with diabetes only; the second meeting consisted of a combination of people with diabetes and HCPs and the third meeting consisted of HCPs only.

### 1.2 Study design

**Figure 1:**
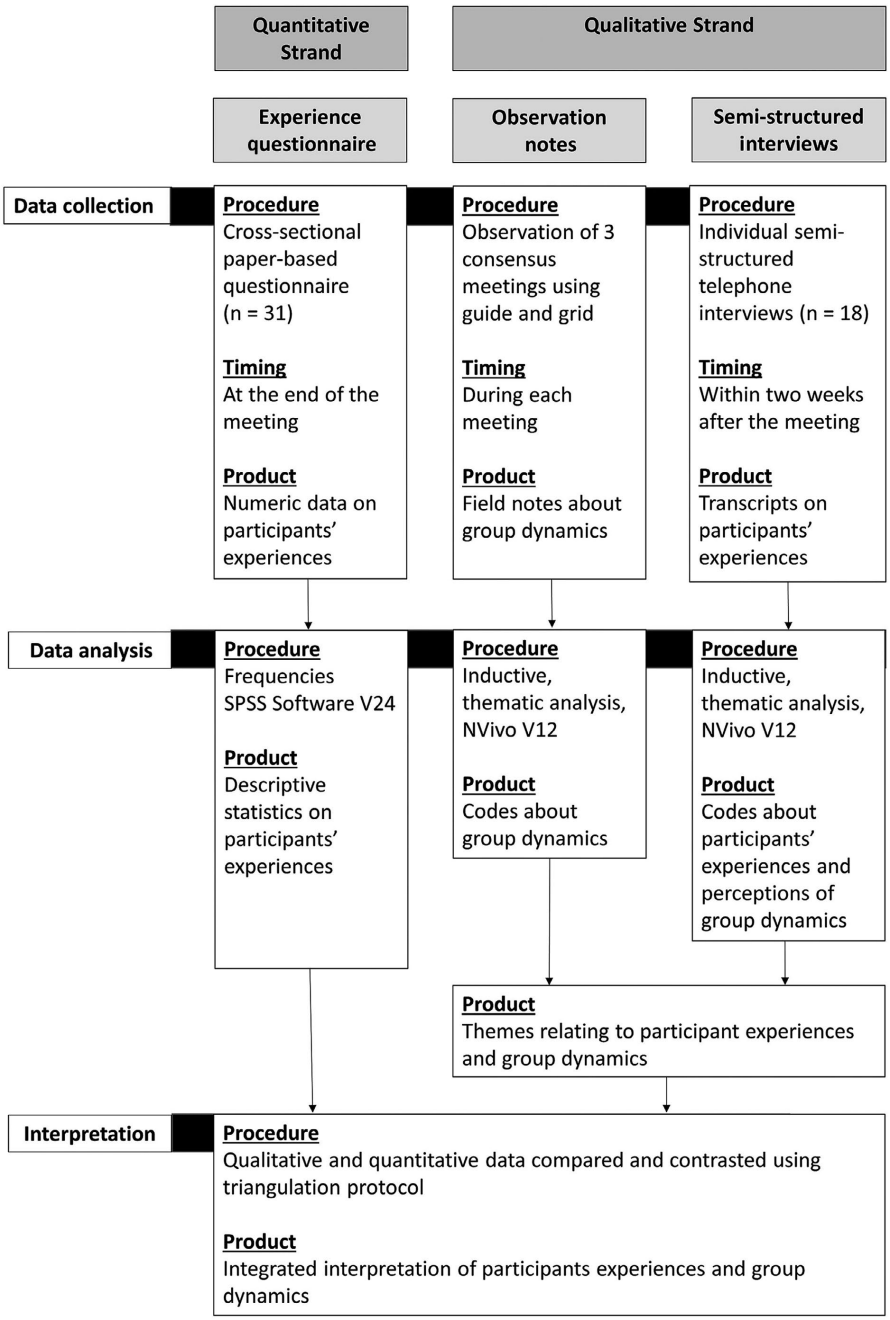
Procedural diagram of the convergent study design

The SWAT used a mixed methods convergent design to understand and compare participants’ experiences of taking part in the consensus meetings (Figure 1). A one‐phase design was used, where quantitative (experience survey) and qualitative (observation notes and semi‐structured interviews) methods were used during the same timeframe and were given equal weight in the analysis.^9^

Quantitative and qualitative data were collected and analysed separately. Results were merged during interpretation (mixed methods phase). A triangulation protocol was used in this phase to compare key concepts identified in each dataset that related to participants’ experiences of taking part in the meetings.^9^, ^10^ The Good Reporting of A Mixed Methods Study (GRAMMS) framework and the Consolidated Criteria for Reporting Qualitative Studies (COREQ) were used to guide reporting of the findings.^11^, ^12^

### 1.3 Recruitment of participants

People with diabetes were recruited using an information flyer developed by the research team and a graphic designer (Supplementary File 1). The flyer was distributed using a range of recruitment strategies previously identified by Vat et al^13^ (Supplementary File 2).

All individuals who contacted the study team about involvement were sent a 26‐item demographic survey (Supplementary File 3 for survey questions and results). The individuals who returned a demographic survey were randomly assigned (using an online random number generator) to the meeting for people with diabetes‐only or the combined meeting.

HCPs were recruited through professional networks known to the SWAT and IDEAs study teams. HCPs were initially sent an email or letter inviting them to take part in the consensus meeting. This was followed by a phone call to confirm their attendance. HCPs were either allocated to the HCP‐only or combined meeting based on their availability to attend.

### 1.4 Semi‐structured consensus meetings

Before the meetings, the IDEAs study team (FR, SMH) developed (a) a short summary of existing evidence on barriers to and enablers of attendance at diabetic retinopathy screening, and interventions to address non‐attendance and (b) a survey asking participants to rate intervention components according to acceptability (like it, think it makes sense) and feasibility (think it can be done). The survey was based on measures developed by Weiner et al^14^ Materials were reviewed by adult literacy experts (Irish National Adult Literacy Agency) and a Patient and Public Involvement (PPI) group from another research project and revised based on their feedback. Before the meeting, the evidence summary and survey was sent to all meeting participants in electronic or paper format depending on participants’ preferences. Survey responses were collated and analysed descriptively by a member of the IDEAs study team (FR) and a summary of the results was prepared to be presented at each meeting.

Each consensus meeting was held from 6.30 to 8.30 pm in University College Cork. Before each meeting (at 6 pm), the lead SWAT researcher (ER) held an informal briefing for people with diabetes on key medical and research terms, the aim of the meeting and their role as patient contributors. Each meeting was facilitated by an experienced facilitator (male). During the meetings, a summary of the survey results was presented to participants, followed by a series of small group discussions facilitated by FR, SMH and EP. Participants were asked how each intervention component would work in practice and which mode of delivery would work best. Each small group was asked to nominate a lead to feed back their discussion to the larger group. Each group discussion was audio recorded.

### 1.5 Quantitative strand

#### 1.5.1 Experience questionnaire

At the end of each meeting, all participants were asked to complete a questionnaire about their experience of the meeting. The objective of the questionnaire was to understand individual experiences of taking part in the meeting, asking them to rate how they felt about their participation and the participation of other group members; how decisions were made by the group; and the potential impact of the decisions that were made. We were unable to find a suitable validated instrument that was appropriate for our questionnaire objective and context (one‐off participatory research process). Therefore, we developed our own questionnaire based on sample items from a non‐validated survey instrument published by Schulz et al^15^ For additional information on the questionnaire development, please see Supplementary File 4. The original phrasing of the sample items was maintained, with the exception of some questions that were changed to statements to fit with a Likert scale format. Agreement with each statement was measured on a 5‐point Likert scale ranging from ‘strongly disagree’ to ‘strongly agree’. The questionnaire also contained an open‐ended comment box for any other comments or suggestions. At the bottom of the questionnaire, participants were invited to ‘opt in’ if they were interested in participating in a follow‐up interview on their experiences of taking part in the meeting.

#### 1.5.2 Quantitative data analysis

Questionnaire responses were entered into SPSS software (version 24) and analysed using descriptive statistics. The five response categories were collapsed into three categories—‘Agree’, ‘Neither agree nor disagree’ and ‘Disagree’.

### 1.6 Qualitative strand

#### 1.6.1 Observation notes

The SWAT lead researcher (ER) observed each consensus meeting and took comprehensive field notes. The objective of the observation was to understand how members participated and interacted with other meeting members and how they made decisions for the development of the intervention (group dynamics and decision‐making processes). An observation guide and grid were used to guide note taking and as a reminder of the events and issues of most importance (Supplementary File 5).^16^ The observation guide contained two overarching questions: ‘How is the group working overall?’ and ‘How is the group making decisions?’. The observation grid contained six constructs informed by group dynamics and decision‐making processes literature.^17^, ^18^, ^19^, ^20^ These constructs were as follows: participation/non‐participation, dominance/submissiveness, in‐groups/out‐groups,^1^An in‐group is a social group to which a person psychologically identifies as being a member. An out‐group is a social group with which a person does not identify. body language and facial expressions, gaze, and effect of expert/lay knowledge. After each meeting, the researcher met with the group facilitators to discuss and document their experiences and perspectives as supplementary information.

#### 1.6.2 Semi‐structured interviews

Within 2 weeks of the consensus meetings, semi‐structured telephone interviews were conducted with the consensus meeting participants who agreed to take part in an interview in the experience questionnaire. The objective of the interviews was to gain insights into individual experiences of taking part in the meeting in terms of: how comfortable they felt in the meeting; how they felt members of the group interacted with each other and how they felt they worked together to make decisions (ie, whether there was agreement, conflict, synergy). Interviews were audio‐recorded (see Supplementary File 6 for Interview Topic Guide). Interviews were conducted by ER, a young female PhD candidate. All participants were familiar with ER as she facilitated the briefing session prior to the consensus meetings. At the beginning of each interview, the SWAT lead researcher (ER) stressed to participants that she was independent to the trial study team that were running the consensus meetings and therefore would not be offended if they described negative experiences.

#### 1.6.3 Qualitative data analysis

Field notes were collated, and audio recordings were transcribed verbatim. All qualitative data were managed using NVivo software (version 12). Thematic analysis was carried out following Braun and Clarke guidelines.^21^ Firstly, an extensive familiarization process was conducted by two researchers (ER, EP), where notes and transcripts were read and re‐read multiple times. ER open coded all the observation notes and transcripts (using semantic and latent codes) and developed three separate sets of codes—one set for each meeting. The pattern and meanings of codes were then examined across the three meetings to identify one set of candidate or potential themes relating to participants’ experiences and group dynamics. Themes were developed using a conventional or ‘bottom‐up’ approach, whereby themes were developed directly from the data.^21^ ER discussed each theme with EP to revise, refine and define themes.

### 1.7 Mixed methods phase

After separate analysis of quantitative and qualitative data (as described above), the data were compared using a triangulation protocol. Triangulation provides a visual and tabular representation of the findings from qualitative and quantitative data, allowing for a clearer comparison and broader interpretation.^22^ The steps taken to create the triangulation protocol are outlined in Table 1 below.

**Table 1:** Steps taken to create triangulation protocol

	Step	Activity
1.	Collate key findings from each dataset	This was done by examining the original data, interpretation and reports of analysis. For quantitative data, each questionnaire item was deemed as a separate key finding. For qualitative data, multiple key findings were identified within each theme, as themes were too broad in their descriptions to compare directly to quantitative findings
2.	Group key findings into concepts	Key quantitative and qualitative findings were grouped together into concepts according to how they related to participants’ experiences and group dynamics (eg freedom of expression, balance of participation)
3.	Create table for triangulation protocol	A table was created with each column representing the data source (questionnaire, observation and interview) and each row representing a key concept
4.	Map key findings to table	Key findings were then mapped to the table to examine where findings from each method agreed (convergence), offered complementary information on the same issue (complementarity), appeared to contradict each other (dissonance) or appeared in one method and not the other (silence)^46^
5.	Explore intermethod discrepancies	This was done by examining the methodological rigour of each method and re‐examining the data in light of the discrepancy^47^

### 1.8 Patient and Public Involvement (PPI) component

A PPI partner (GF) was involved in the SWAT from the outset. The PPI partner is a person with diabetes, previously known to the lead author (ER). She contributed to the initial discussions about the study which ultimately informed the SWAT grant application, reviewed the application and made changes to its content. GF was also involved in the development of materials used to recruit PPI contributors and assisted the research team with recruitment by posting recruitment flyers online via social media networks. In addition, she contributed to and reviewed each draft of this manuscript and is a co‐author on this publication.

### 1.9 Ethics

The study received ethical approval from the Social Research Ethics Committee (SREC) at University College Cork. Written informed consent was obtained from all participants prior to taking part in the consensus meetings and completing the questionnaire. Telephone consent was obtained from participants prior to taking part in the interviews.

## 2 RESULTS

### 2.1 Participants

**Figure 2:**
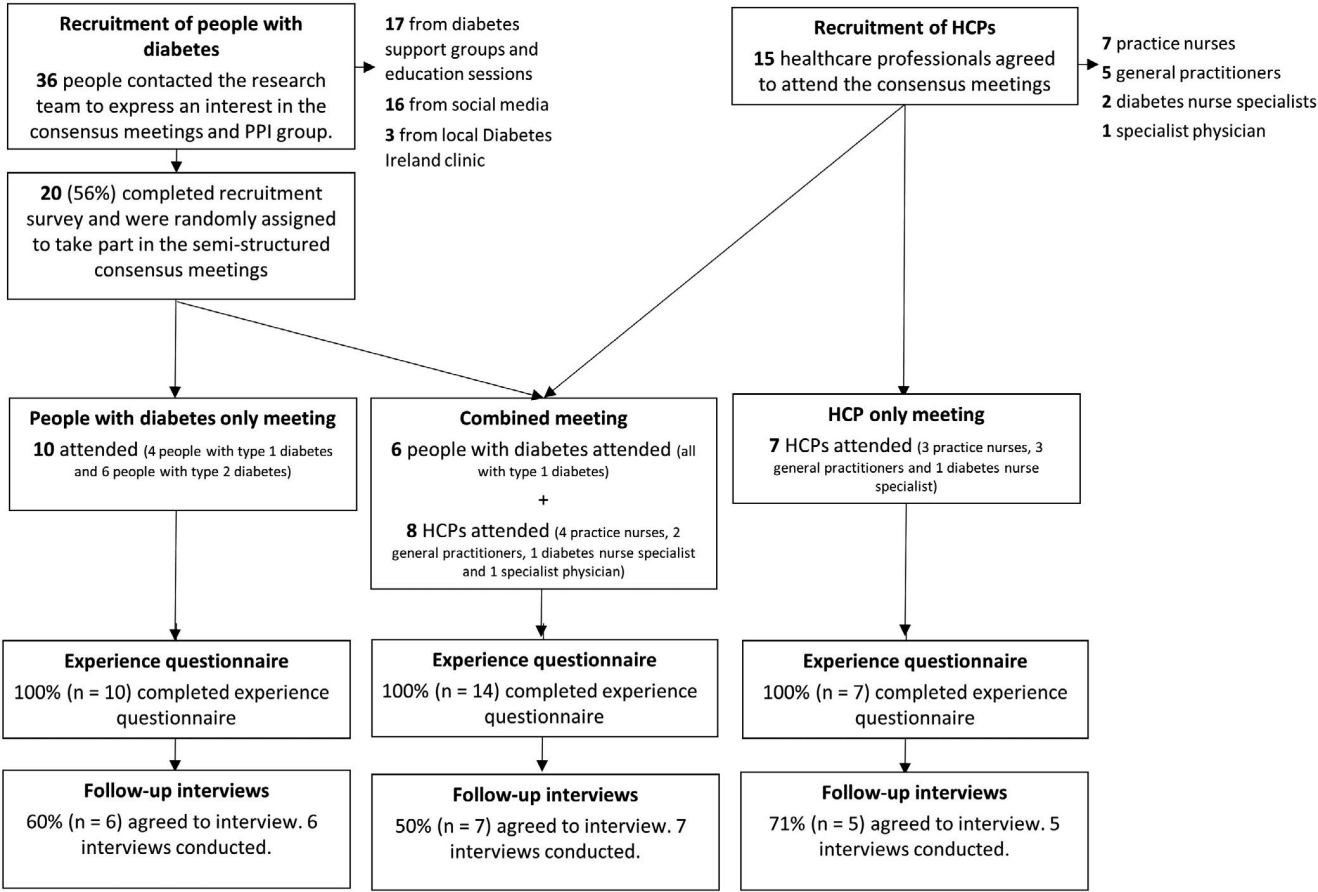
Flow diagram of recruitment and response rates

A total of 36 people contacted the research team expressing an interest in the SWAT. Of these, 20 completed the recruitment survey (see Supplementary File 3 for recruitment survey results). These 20 people were randomly assigned to either the people with diabetes‐only meeting (4 with type 1 diabetes and 6 with type 2 diabetes) or the combined meeting (6 with type 1 diabetes, 3 with type 2 diabetes and 1 carer). All 10 people attended the people with diabetes‐only meeting (attendance rate 100%) and 6 people with diabetes attended the combined meeting (attendance rate 60%). An invitation to attend was sent out to 50 HCPs (practice nurses, diabetes nurse specialists, general practitioners and specialist physicians), of whom 8 attended the combined meeting and 7 attended the HCP‐only meeting (attendance rate 30%). Further details on the recruitment and response rates for each stage of the data collection are shown in Figure 2 below.

### 2.2 Quantitative results

All consensus meeting participants (n = 31) completed the experience questionnaire (response rate 100%). Table 2 shows the results of the questionnaire stratified by meeting type (people with diabetes only, combined and HCP only). The descriptive statistics presented in Table 2 demonstrate that there were no differences in participants’ self‐reported experiences of the three meetings. All participants across the three groups agreed with the statements ‘*I felt comfortable expressing my opinion in the group’*, *‘I felt my opinions were listened to and considered by other group members’* and ‘*I did not feel pressured to go along with the decisions of the group even though they did not agree*’. Some participants agreed with the statements that ‘*I thought that certain individuals spoke more than others in the group’* and ‘*I felt that certain individuals had more influence over the decision‐making process than others’*. A number of participants expressed doubt that they could influence the decisions made during the meeting.

**Table 2:** Results of the participant experience questionnaires stratified by meeting type

Item	Meeting	Agree N (%)	Disagree N (%)	Neither agree nor disagree N (%)
I felt comfortable expressing my opinion in the group	People with diabetes	10 (100)	‐	‐
	Combined	14 (100)	‐	‐
	HCP	7 (100)	‐	‐
I felt my opinions were listened to and considered by other group members	People with diabetes	10 (100)	‐	‐
	Combined	14 (100)		‐
	HCP	7 (100)	‐	‐
I felt part of the group (like I belonged to the group)	People with diabetes	10 (100)	‐	‐
	Combined^a^	12 (92.3)	‐	1 (7.7)
	HCP	7 (100)	‐	‐
I felt pressured to go along with the decisions of the group even though I did not agree	People with diabetes	‐	10 (100)	‐
	Combined	‐	14 (100)	‐
	HCP	‐	7 (100)	‐
I felt a sense of trust and openness between group members	People with diabetes	10 (100)	‐	‐
	Combined	13 (92.9)	‐	1 (7.1)
	HCP	7 (100)	‐	‐
I thought that certain individuals spoke more than others in the group	People with diabetes only	3 (30)	6 (60)	1 (10)
	Combined	4 (28.6)	6 (42.8)	4 (28.6)
	HCP	3 (42.9)	3 (42.9)	1 (14.2)
I felt that I could influence the decisions made by the group	People with diabetes	7 ((70)	‐	3 (30)
	Combined	8 (57.1)	1 (7.1)	5 (35.7)
	HCP^a^	4 (66.7)	‐	2 (33.3)
I felt that certain individuals had more influence over the decision‐making process than others	People with diabetes	3 (30)	6 (60)	1 (10)
	Combined	2 (14.3)	9 (64.3)	3 (21.4)
	HCP	1 (14.3)	3 (42.9)	3 (42.9)
I have increased my knowledge about important topics since participating in this group	People with diabetes^a^	8 (88.9)	‐	1 (11.1)
	Combined	10 (71.4)	1 (7.1)	3 (21.4)
	HCP	6 (85.7)	‐	1 (14.3)
By working together, we can influence decisions that affect the research process	People with diabetes only	10 (100)	‐	‐
	Combined	13 (92.9)	‐	1 (7.1)
	HCP	7 (100)	‐	‐
By working together, we can influence decisions that affect people with diabetes	People with diabetes	10 (100)	‐	‐
	Combined	14 (100)	‐	‐
	HCP	7 (100)	‐	‐

^a^ Missing data.

### 2.3 Qualitative results

In total, 18 questionnaire respondents agreed to be contacted for a follow‐up interview. Interviews were conducted with participants from the people with diabetes‐only (n = 6), combined (n = 7) and HCP‐only (n = 5) meetings. Interviews were, on average, 34 minutes in duration (range 18‐56 minutes).

Four themes were developed from the qualitative data relating to participants’ experiences and group dynamics: perceived lack of common ground; feeling empowered versus undervalued; needing to feel safe and going off task to fill the void.

### 2.4 Perceived lack of common ground

In the people with diabetes‐only meeting, there were differences between participants in terms of diabetes type, length of diagnosis and education level. In the HCP‐only meeting, differences included profession (eg medical doctor, practice nurse, diabetes nurse specialist), experience of working with people with diabetes, and size, location and nature of their practices. During the interviews, participants from these two meetings described these demographic, geographical and clinical differences as ‘*small’* differences, which they welcomed as they felt it allowed them to bring different perspectives to the topics they were discussing. They focused on the common ground they shared with other meeting participants and identified with one another based on the shared experience of living with diabetes or caring for people with diabetes. They felt that they were all ‘*singing from the same hymn sheet’* (P3, person with diabetes, people with diabetes‐only meeting) and described being able to come together to make decisions that incorporated different perspectives:

It was interesting to hear their views. We were all on the same page, but we were coming from different angles and we used that then; we came together and made the decisions together. ( P2 , person with diabetes, person with diabetes‐only meeting)

In contrast, a lack of common ground was reported by participants in the combined meeting. This created a division in the group, a ‘them’ and ‘us’ attitude, which was evident in the interview and observation data. In the interview data, people with diabetes stated that there was *a ‘complete clash of perspectives’* (P9, person with diabetes, combined meeting) between people who lived with the condition and HCPs who cared for people with diabetes. HCPs reported that people with diabetes and HCPs were ‘*two different sides of the divide’* (P11, HCP, combined meeting). The observation data also suggested a division between people with diabetes and HCPs in the combined meeting. At the beginning of the meeting, people with diabetes and HCPs sat on opposite sides of each small table. During the small group discussions, participants expressed their opinions as collective opinions of their stakeholder group. Rather than expressing individual opinions (eg ‘I think that…’or ‘My experience is…’), people with diabetes spoke on behalf of all the people with diabetes in the group, and HCPs spoke on behalf of all HCPs in the group (eg ‘We feel that… don't we?’ and ‘As people with diabetes, we think that…’). Moreover, during the small group discussions, each stakeholder group focused their gaze on the other stakeholder group, resulting in people with diabetes and HCPs talking at each other, at opposite sides of each table. This was in contrast to the people with diabetes‐only and HCP‐only meeting, where members focused their gaze on all members around the table.

Participants’ lacking a sense of shared experience was accompanied by differences in perceptions around the balance of participation. During all three meetings, it was observed that some participants spoke more frequently and for longer than others. In the interviews, participants from the people with diabetes‐only and HCP‐only meetings perceived this unbalanced participation as a natural consequence of any group dynamic. They mainly attributed it to different personalities. In contrast, HCPs from the combined meeting attributed the unbalanced participation to people putting too much emphasis on their own personal experiences:

It was very much centred around them [people with diabetes] and a lot of the offerings that I had in terms of experience were nothing in comparison to what they felt as people that have the problem. Which is fine. But that wasn’t really the point. The point is that I don’t have diabetes, that is not my personal experience. But I am still the one left in the room everyday trying to deal with patients… But I just couldn't come out with it on the night. I just didn't. It wasn't going to be heard. ( P12 , HCP , combined meeting)

### 2.5 Feeling empowered versus undervalued

In the interviews, participants from the people with diabetes‐ and HCP‐only meetings reported learning from other meeting members and feeling empowered by the event. In the people with diabetes‐only meeting, participants stated that they learned from one another about how they can better manage their condition and about the difference between type 1 and type 2 diabetes. Those who had been diagnosed with diabetes for a long time described gaining a renewed compassion for those who were newly diagnosed. Participants from the HCP‐only meeting reported learning about the importance of encouraging their patients to attend screening, about the roles of different HCPs and about the cultural difficulties and language barriers that some practices face due to a high number of non‐English speaking patients.

There were also some reports of learning in the combined meeting. People with diabetes said they gained a new insight into the work practices of HCPs—in particular, the increased workload experienced by HCPs. The HCPs reported gaining an insight into the struggles of having to live with a medical condition:

I put in a couple of thousand eye drops a year, it doesn't mean anything to me like. But it obviously means something for patients who are having to go through this – and you know the awkwardness of getting appointments and driving to and from appointments and getting a lift and all that side of things. ( P14 , HCP , combined meeting)

However, participants from the combined meeting reported feeling undervalued by the other stakeholder group. People with diabetes felt that HCPs did not understand how it feels to live with a chronic illness, describing ‘*a complete clash of the reality of living with diabetes versus a medical professional's perspective’* (P7, person with diabetes, combined meeting). Among some of the HCPs, there was a sense that any contributions they made during the meeting were not valued by people with diabetes because the experience of living with diabetes was deemed more important than the experience of caring for people with diabetes:

I've worked in four different GP practices at this stage and all very different. And yet I felt like as if any value that I had to add to the conversation was kind of almost either misheard or not really heard, or almost felt not quite as relevant because of their personal experiences. Which is fair enough. But that was not what the meeting was about. ( P13 , HCP , combined meeting)

### 2.6 Needing to feel safe to express honest opinions

In the interviews, participants from the people with diabetes‐only and HCP‐only meetings reported an open, honest, relaxed and non‐judgemental environment, where everyone had a voice and was heard. This environment made participants feel safe and comfortable to express their opinions. They also indicated that the small group discussions added to their feelings of safety as people who do not like speaking in public felt less intimidated about expressing their opinions:

I’m not one really for expressing my opinions. I am kind of … I wouldn’t put my hand up the first time, let’s say. But I did feel very comfortable expressing my opinion in the small group. ( P15 , HCP , HCP ‐only meeting)

Conversely, participants from the combined meeting reported feeling uncomfortable and unable to express their opinions as they were conscious of the other stakeholder group in the room. Both people with diabetes and HCPs said they felt they had to ‘*hold back’* their opinions. People with diabetes reported feeling that they could not be honest about the ‘*non‐compliant’* (P9, person with diabetes, combined meeting) aspects of managing their diabetes as the HCPs may judge them for it:

I don’t think when you are sitting at a table with HCPs that you’re going to be discussing the non‐compliant things you do… It’s probably not the best environment, let’s say, to get out some of the smaller things that people do that may not be approved by the other group in the room. ( P8 , person with diabetes, combined meeting)

On the other hand, HCPs were conscious of confidentiality issues: they were concerned that if they mentioned a particular case, people with diabetes could potentially identify who that patient was, since *‘[this location] is a very small place’* (P11, HCP, combined meeting):

I felt a bit kind of reticent about how free [I could talk about my experiences as a healthcare professional]… It’s different when you are divulging, you know, work practices and difficulties and challenges and personal experiences at work, when it is other medical professionals. But when you have effectively patients there, it is like a big difference. ( P13 , HCP , combined meeting)

In addition, the HCPs indicated that they did not feel comfortable talking about the service that they worked in as they felt anxious that people with diabetes would confront them on the long waiting times or other issues they had with that particular service.

### 2.7 Going off task to fill the void

Analysis of interview data indicated that participants across all three meetings felt they were able to work together. They reported that the content for discussion was relevant to them as users and providers of health services.

However, the observation data show that although members of the combined meeting appeared to work together, both stakeholder groups were defensive about what intervention components would not work and at times in the meeting nothing seemed feasible. This resulted in each stakeholder group feeling uncomfortable in asserting what they felt the other group should or should not do. To fill this void, participants began to go off task as they focused their discussions on the ‘other’. The ‘other’ took different forms throughout the meeting: the screening service, people with diabetes who were not in the room (eg those with type 2 diabetes), and funding and resource limitations in general practice. Even though they were being asked to discuss and make recommendations on how the intervention would work in primary care, the combined meeting participants resorted to making recommendations about how screening uptake could be increased on a national basis through nationwide TV and radio campaigns.

### 2.8 Mixed methods results

The results of the mixed methods analysis are presented in Table 3. Six key concepts relating to participants’ experiences and group dynamics were identified from the datasets: freedom of expression; understanding and respect; balance of participation; learning; productive collaboration and group cohesion. When key findings were mapped to the overarching concepts, there were four instances of dissonance (where data appeared to contradict each other), two instances of convergence (where data agreed) and two instances of complementarity (where data offered complementary information on the same issue). There were no instances of silence (where data appeared in one method and not in the other).

**Table 3:** Results of mixed methods analysis (triangulation protocol)

Key concept	Quantitative strand	Qualitative strand		
	Questionnaire	Observation notes	Interviews Interviews	
Freedom of expression	In all three meetings, participants were comfortable expressing their opinions and felt a sense of trust and openness between group members	In the combined meeting, participants did not appear to be comfortable asserting what the other stakeholder group should/should not be doing	In the people with diabetes‐only and HCP‐only meetings, participants reported that it was an open, honest and relaxed environment where they felt comfortable expressing their opinions In the combined meeting, participants reported feeling uncomfortable and unable to express their opinions as they were conscious of the other stakeholder group in the room	Dissonance
Understanding and respect	In all three meetings, participants felt their opinions were listened to and considered by other group members, and that they could influence the decisions being made by the group	‐	In the combined meeting, participants reported feeling undervalued by the other stakeholder group	Dissonance
Balance of participation	In all three meetings, some participants felt that certain individuals spoke more than others and had more influence over the decision‐making process	In all three meetings, some participants spoke more frequently than others and for longer lengths of time	In the people with diabetes‐only and HCP‐only meetings, participants were understanding of the unbalanced participation and saw it as a natural consequence of any group dynamic In the combined meeting, HCPs attributed unbalanced participation to people putting too much emphasis on their own personal experiences	Convergence, complementarity
Learning	In all three meetings, most participants felt they increased their knowledge as a result of attending	In all three meetings, participants appeared keen to learn from one another as they asked each other about their experiences	In all three meetings, participants reported learning from one another and provided specific examples of this learning	Convergence, complementarity
Productive collaboration	In all three meetings, participants reported that they were able to work together to influence decisions that affect the research process and people with diabetes	In the combined meeting, although participants appeared to work together, each stakeholder group did not make any comments on what the other stakeholder group should/should not do. Instead, they made recommendations that were not relevant to the intervention (unproductive collaboration)	In all three meetings, participants reported being able to work together as they felt the content for discussion was relevant to them as users and providers of health services	Dissonance
Group cohesion	In all three meetings, participants reported they were part of the group (like they belonged to the group)	In the combined meeting, it was evident that there was a division between both stakeholder groups (eg both groups spoke at each other across the each table as opposed to with each other around each table).	In the people with diabetes‐only and HCP‐only meetings, participants reported that there were some ‘small’ differences between meeting members, but added that this was a good thing as it allowed them to bring different perspectives to the topics they were discussing In the combined meeting, people with diabetes reported that there was a ‘complete clash of perspectives’ between people with diabetes and HCPs; HCPs reported that people with diabetes and HCPs were ‘two different sides of the divide’	Dissonance

The four instances of dissonance between quantitative and qualitative data were wholly due to the fact that in the questionnaire participants reported positive experiences of taking part in the meetings, whereas the observation and interview data highlighted some negative experiences and divergent opinions. For example, in relation to *freedom of expression*, the questionnaire data showed that in all three meetings, participants reported feeling comfortable expressing their opinions and reported a sense of trust and openness between group members. In the observation data, participants in the combined meeting did not appear to be comfortable asserting what the other stakeholder group should/should not do as part of the intervention. Furthermore, in the interviews participants reported feeling uncomfortable and unable to express their opinions as they were conscious of the other stakeholder group in the room.

The instances of complementarity were largely due to the design of the data collection tools. The questionnaire items were designed to be concise and did not require the participants to give any additional details. Whereas in the interviews, participants had the opportunity to expand and give more detail. For example, in the key concept *learning*, the questionnaire item asked participants to indicate how much they agreed with the statement ‘I have increased my knowledge about important topics since participating in this group’, whereas in the interviews participants had the opportunity to expand and give specific examples of what they had learned (eg people with diabetes learned how they can better manage their condition, HCPs leaned about the importance of encouraging their patients to attend screening, etc).

## 3 DISCUSSION

### 3.1 Summary of key findings

The aim of this study was to compare participants’ experiences of taking part in the three consensus meetings. The results of the questionnaire suggested that participants had largely positive experiences of taking part in the consensus meetings and there were no differences in participants’ experiences between the three meetings. However, results of the observation and interviews highlighted that participants in the combined meeting had different experiences from those in the other two meetings. The perceived lack of common ground between people with diabetes and HCPs in the combined meeting led participants to feel undervalued by the other stakeholder group as they felt that the other group did not understand their perspective. Participants in the combined meeting were reluctant to express their opinions and were defensive about what would/ wouldn't work in terms of developing the intervention. As a result participants in the combined meeting went off task and made recommendations which were not entirely relevant for the intervention. In this study, involving patients and HCPs simultaneously in a consensus process was not found to be as suitable as involving each stakeholder group separately.

### 3.2 Links to existing literature

In the people with diabetes‐only and the HCP‐only meetings, participants welcomed their diversity as it allowed them to hear different perspectives on the topics they were discussing. This finding is consistent with existing literature, with many theorists arguing that knowledge diversity can improve group performance by enhancing a group's ability to be creative and to discover novel solutions.^23^, ^24^, ^25^ In these meetings, participants focused on their common ground and described being able to come together to make decisions that incorporated a range of perspectives. Previous research suggests that congruent groups—ie when group members are socially tied and share the same information—are more likely to be productive and successful.^26^

The perceived lack of common ground between people with diabetes and HCPs in the combined meeting created a ‘them’ and ‘us’ scenario, with participants reluctant to express their opinions. This raises questions about whether too much difference within groups is counterproductive or divisive. Existing research on the productivity of incongruent groups—ie when social and knowledge subgroups are present within a group has found that subgroups can create a divide between group members, undermining the groups’ ability to work together and be productive.^26^

Some HCPs in the combined meeting felt their contributions were not valued by people with diabetes because the experience of living with diabetes trumped the experience of caring for people with diabetes. This finding may reflect the changing nature of the patient/HCP relationship over the last 20 years—from a paternalistic model where the patient seeks help and is compliant to the professional who makes the decisions, to a more patient‐centred approach.^27^ This approach expects HCPs to enter the patient's world and to see the illness through the patient's eyes.^27^ This prioritization of the patient experience has benefited patient outcomes.^28^ However, as HCPs are often responsible for delivering interventions, their perspectives in the intervention development process are crucial for maximizing intervention feasibility. Involving multiple users in the intervention development process is not about understanding which perspective is more valid or more important, it is about understanding all the different perspectives so that the intervention is more acceptable, engaging and feasible to implement.

### 3.3 Strengths and limitations

One of the strengths of this study was the use of a mixed methods, convergent design which produced a more complete understanding of participants’ experiences and group dynamics. It also allowed for the cross‐validation of findings from each method resulting in more substantiated findings than sequential designs or quantitative or qualitative approaches alone.^9^ The qualitative theme ‘needing to feel safe’ may explain the instances of dissonance between quantitative and qualitative data as participants completed the questionnaire at the end of each meeting while they were still sitting close to other participants. Some small groups even filled out the questionnaire together. As a result, participants may not have felt comfortable voicing concerns. In the interviews, on the other hand, participants may have felt safer in a one‐to‐one environment with a researcher who they were already familiar with. The fact that the researcher stressed that she was independent to the consensus meeting research team and her informal approach may have made them more comfortable to speak openly about their experiences of taking part in the meeting. The timing of the questionnaire may have also played an important role. The questionnaire was handed out at the end of the meeting, late in the evening. Participants may have been eager to get home and they may not have fully thought about the responses they were providing. However, in the interviews, participants had time to reflect on their experiences and provide a more comprehensive account as a result. This is consistent with Krosnick's theory of survey satisficing which is based on the assumption that optimal survey completion involves doing a great deal of cognitive work, so if the participant is not fully motivated to complete the survey, he or she is likely to offer responses that seem reasonable and easy to defend.^29^ Although questionnaires are a frequently used tool to evaluate consensus meetings, our findings suggest that they may not always provide a comprehensive assessment of participants’ experiences. This is consistent with a number of previous studies on evaluating participant experiences.^30^, ^31^, ^32^

This study is not without limitations. First, the questionnaire that was used to understand participants’ experiences was based on non‐validated questionnaire items. We were unable to conduct exploratory factor analysis to validate our questionnaire as our sample size did not meet the minimum criteria of 10 participants per questionnaire item.^33^ However, given the increasing importance of evaluating PPI and other participatory research activities,^34^ the questionnaire could be a useful tool in future studies which aim to understand stakeholders’ experiences in similar participatory research contexts. Use of the questionnaire in future studies may allow for reliability testing and validation to be carried out.^35^, ^36^

Second, although the experience questionnaire suggested that there were no differences in participants’ experiences between the three meetings, due to the number of participants, there was limited power to detect a difference (n = 31). Thus, the comparison of participants’ questionnaire responses between the groups is used as only an indicator of participants’ experiences. Given the small sample, we cannot rule out the possibility that differences between the groups could be detected had a larger sample size been used.

Despite using a range of strategies to recruit a representative sample of people with diabetes, another potential limitation of this study was the absence of people with type 2 diabetes in the combined meeting. As the attendance rate of people with diabetes at the combined meeting (60%) was much lower than the people with diabetes‐only meeting (100%), it is plausible that people with type 2 diabetes did not attend because they knew there would be HCPs attending. Existing research has established that people with type 1 and type 2 diabetes have different experiences when managing their condition and engaging with HCPs.^37^, ^38^, ^39^ Therefore, the involvement of people with type 2 diabetes in the combined meeting could have potentially changed the nature of the relationship between patients and HCPs and led to different participant experiences and group dynamics.

Finally, participants were given a choice to participate in an in‐person or telephone interview. All participants chose telephone interviews due to time constraints and location convenience. This could be another potential limitation as researchers have previously expressed concerns about whether telephone interviews are appropriate for qualitative research.^40^, ^41^ These concerns are largely due to the absence of visual cues which may result in the loss of informal communication and contextual information, the inability to develop rapport or to probe and the misinterpretation of responses.^41^ In this study, the quality of telephone data cannot be compared with in‐person data as no in‐person interviews were conducted. However, the researcher had considerable experience conducting phone interviews, maintained a friendly and engaging tone throughout and as mentioned previously, participants were found to be open and frank about their experiences.

### 3.4 Implications

The results of this study provide much needed evidence on how different ways of involving patients and health‐care professionals can lead to differing participant experiences and group dynamics. Patient and public involvement (PPI) in research is increasingly becoming a requirement in health research and for many research funders. INVOLVE, a national advisory body funded by the National Institute for Health Research (NIHR) in the UK, defines public involvement as research being carried out ‘with’ or ‘by’ members of the public rather than ‘to’, ‘about’ or ‘for’ them.^42^ In this study, the lines between research participation and involvement were blurred, as is often the case with PPI.^43^ People with diabetes were research participants in the consensus meetings, experience questionnaire and semi‐structured interviews. However, their role in the consensus meeting was to discuss and make decisions about the intervention content and mode of delivery which could be viewed as PPI.^44^, ^45^ This study shows that the context and nature of involvement can have important implications for its impact. These findings are not only relevant to health intervention researchers but to all individuals interested in involving patients and members of the public in health research, policy and in the planning and development of health care more broadly.

## 4 CONCLUSION

Although the results of the experience questionnaire showed no differences in participants’ experiences across the three meetings, the results of the observation and interviews highlighted that participants in the combined meeting had different experiences. In this study, involving patients and HCPs simultaneously in a consensus process was not found to be as suitable as involving each stakeholder group separately. The study provides much needed evidence on how different ways of involving patients and health‐care professionals can lead to differing participant experiences and group dynamics.

## CONFLICT OF INTEREST

The authors have no conflicts of interest.

## Supporting information

Supplementary File 1

Supplementary File 2

Supplementary File 3

Supplementary File 4

Supplementary File 5

Supplementary File 6

## Data Availability

The data that support the findings of this study are available on request from the corresponding author. The data are not publicly available due to privacy or ethical restrictions.
