# Supplementary material for: 'It just wasn’t going to be heard’: A mixed methods study to compare different ways of involving people with diabetes and health‐care professionals in health intervention research
Source: Health Expect. 2020 May 1;23(4):870–83. doi: 10.1111/hex.13061 (PMC7495083; doi:10.1111/hex.13061)

Supplementary File 1: PPI Recruitment Flyer

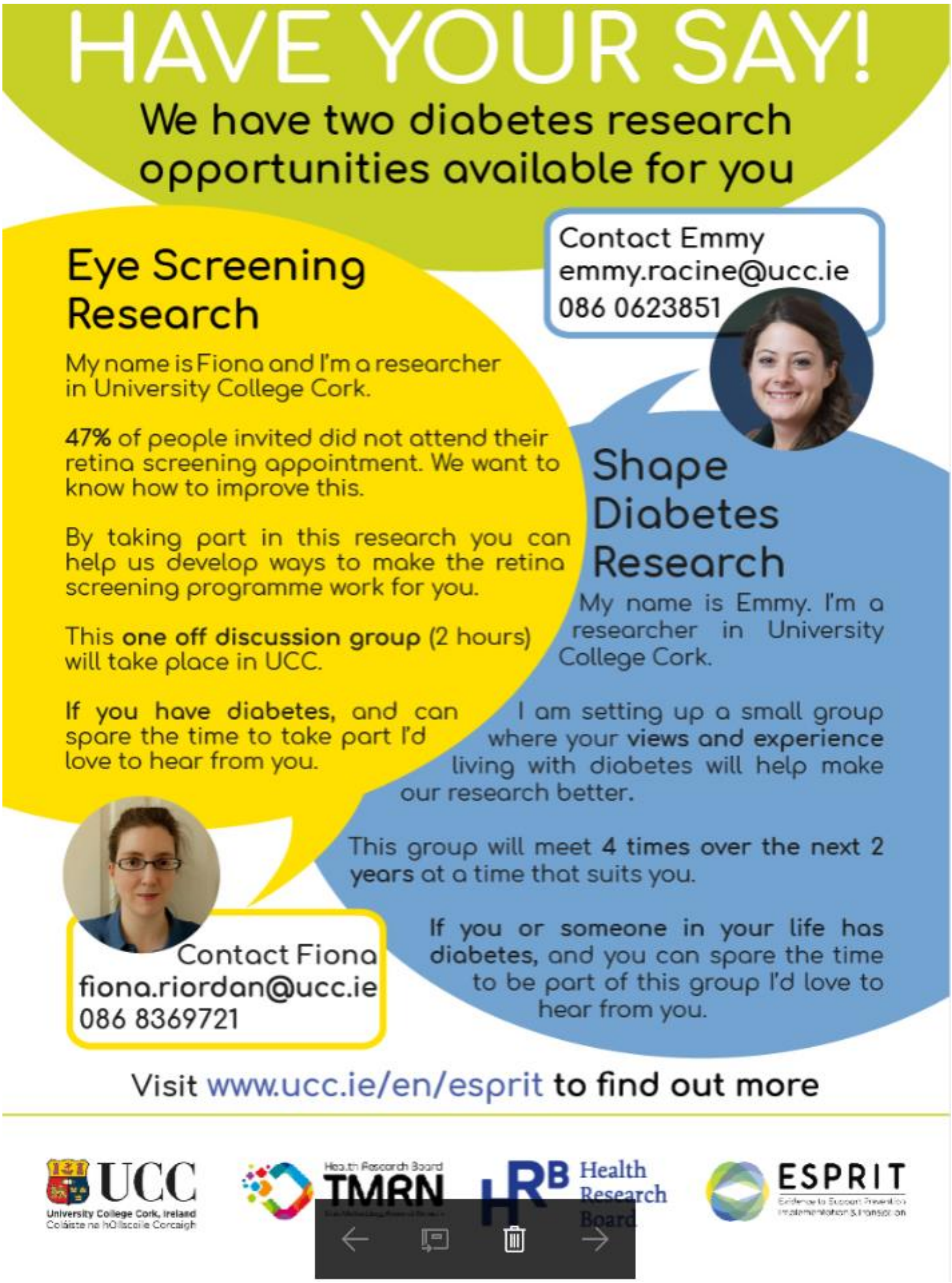

Supplement: Supplementary file 1 [file HEX-23-870-s001.pdf]
