# Supplementary material for: 'It just wasn’t going to be heard’: A mixed methods study to compare different ways of involving people with diabetes and health‐care professionals in health intervention research
Source: Health Expect. 2020 May 1;23(4):870–83. doi: 10.1111/hex.13061 (PMC7495083; doi:10.1111/hex.13061)
Supplement: Supplementary file 2 [file HEX-23-870-s002.pdf]

Supplementary File 2: PPI Recruitment Strategies

The information flyer was distributed over an 8 week period from 11/08/18 – 11/10/18. The strategies used to circulate the flyer included social marketing (e.g. social media), community outreach (e.g. community and religious groups), health system (e.g. GP practices and hospital waiting rooms), and partnering with community and advocacy organisations (e.g. national organisations and educational institutions).

Recruitment strategy	Details	Number	Timing	Response (n)
**Social marketing recruitment**	We asked our PPI partner (diabetes advocate and administrator on 'Diabetes in Ireland' facebook support group) to post the information flyer on facebook and twitter.	4 social media posts	17/08/18-03/09/18	16
	We posted the information flyer on our research team twitter page (@ESPRIT_UCC).	3 social media posts	12/09/18-08/10/18	
**Community outreach recruitment**	We circulated the information flyer to local community and religious groups online and asked them to advertise on newsletters etc.	11 'Mens shed' initiatives<br><br>6 religious groups	05/10/18<br><br>06/10/18	0
**Health system recruitment**	We left information flyers in GP practices, hospital waiting rooms and local diabetes clinic (on tables and noticeboards).	8 GP practice waiting rooms<br>1 hospital waiting rooms	31/08/18-11/10/18 (6 weeks)	3
	We spoke about our research (5 minute overview) at diabetes support groups and	1 diabetes support groups	18/09/18<br><br>12/09/18 + 14/09/18	17

	distributed information flyers to attendees.	2 diabetes education sessions		
**Partnering with other organisations**	We contacted a number of different organisations and asked them to circulate our information flyer to their email list and on their websites.	5 national organisations and educational institutions	27/08/ 2018<br>05/09/2018	0
