# Supplementary material for: 'It just wasn’t going to be heard’: A mixed methods study to compare different ways of involving people with diabetes and health‐care professionals in health intervention research
Source: Health Expect. 2020 May 1;23(4):870–83. doi: 10.1111/hex.13061 (PMC7495083; doi:10.1111/hex.13061)
Supplement: Supplementary file 3 [file HEX-23-870-s003.pdf]

Supplementary File 3: PPI Recruitment Survey Results

Table 3.1: PPI Recruitment Survey Results

Variable	Total (N=20)	%
**Age**		
25-44	5	25
45-64	9	45
65-84	6	30
**Gender [Female]**	12	60
Female		
Male	8	40
**Location**		
Urban	14	70
Rural	6	30
**Healthcare cover**		
Full Medical Card	7	35
GP Visit Card	1	5
Private Health Insurance	13	65
**Nationality and Ethnicity**		
White Irish	20	100
**Education***		
Junior Certificate/ Intercert	1	5.3
Apprenticeship	3	15.8
Leaving Certificate	5	26.3
Diploma	2	10.5
Undergraduate Degree	4	21.1
Masters Degree	2	10.5
Doctorate	2	10.5
**Marital status***		
Single	2	10.5
Married	14	73.7
Separated	1	5.3
Widowed	2	10.5
**Diabetes**		
Type 1	10	50
Type 2	9	45
No diabetes	1	5
**Diabetes diagnosis***		
< 12 months	4	21.1
1-5 years	8	42.1
5-10 years	1	5.3
10+ years	6	31.6
**Research Experience**		
Previous participation in research	6	30
Previous involvement in research	3	15
**Diabetes support and education experience**		
Previous attendance at diabetes support group	16	80
Previous attendance at diabetes education session	9	45
**Diabetic Retinopathy Screening**		
Familiar with term 'diabetic retinopathy screening'	19	95
Attended diabetic retinopathy screening*	18	94.7
Attended screening at hospital**	3	16.7
Attended screening at RetinaScreen provider**	11	61.1
Attended screening at local optician**	4	22.2

**Other health conditions**		
Heart Disease	7	35
Asthma	2	10
Arthritis	4	20
Any emotional or psychiatric problems (such as depression or anxiety)	2	10
Stomach ulcers	1	5
**Attitude to medical appointments**		
I always attend	20	100

*N=19

**N=18
