# Supplementary material for: 'It just wasn’t going to be heard’: A mixed methods study to compare different ways of involving people with diabetes and health‐care professionals in health intervention research
Source: Health Expect. 2020 May 1;23(4):870–83. doi: 10.1111/hex.13061 (PMC7495083; doi:10.1111/hex.13061)
Supplement: Supplementary file 4 [file HEX-23-870-s004.pdf]

Supplementary File 4: Experience Questionnaire

4.1.Additional Information on questionnaire development

Existing validated measures that were deemed unsuitable included questionnaires that measured group dynamics in workplace and organizational settings where participants worked together on an on-going basis (1-3) and a questionnaire that measured participants' experiences of being a research subject (as opposed to being actively involved in a participatory research process)(4). Therefore, we developed our own questionnaire based on sample items from a non-validated survey instrument published by Schulz et al. (5).These questionnaire items were deemed suitable for our research objective and context as they were developed to evaluate individual experiences of group dynamics and short-term measures of partnership effectiveness within community-based participatory research partnerships. Schulz et al. provided 90 sample questionnaire items arranged into 15 categories were developed based on a review of the group dynamics literature, previous instruments (where available) and extensive input from community stakeholders involved in three separate participatory research partnerships. The sample questionnaire items were designed to be used selectively based on their relevance to the topics being evaluated and not combined together as one single instrument. As there were some overlap between items and some items were not relevant to our study objective, we selected 11 items from seven categories. This helped to ensure the questionnaire was relatively short and straightforward to complete at the end of the two-hour consensus meetings (6). Categories included were: (1) comfort level for expressing opinions: communication, (2) level of influence and power of self and others in the group, (3) perceived level of trust, (4) personal, organizational and community benefits of participation, (5) sense of ownership/ belonging to the group: cohesion, (6) group empowerment and (7) community empowerment. Categories excluded were: (1) Leadership and participation, (2) How well the group recognizes and addresses conflicts and problems, (3) Decision-making procedures, (4) Problem solving processes, (5) Meeting organisation, agenda setting, facilitation and staffing, (6) Accomplishments/impact of the group and (7) Member background and meeting attendance.

4.2. Experience Questionnaire

EXPERIENCE SURVEY

	**Please indicate how much you agree with each statement:**	**Strongly Disagree**	**Disagree**	**Neither Agree Nor Disagree**	**Agree**	**Strongly Agree**
1.	I felt comfortable expressing my opinion in the group.					
2.	I felt my opinions were listened to and considered by the other group members.					
3.	I felt part of the group (like I belonged to the group).					
4.	I felt pressured to go along with the decisions of the group even though I did not agree.					
5.	I felt a sense of trust and openness between group members.					
6.	I thought that certain individuals spoke more than others in the group.					
7.	I felt that I could influence the decisions that the group made.					
8.	I felt that certain individuals had more influence over the decision-making process than others.					
9.	I have increased my knowledge about important topics since participating in this group.					
10.	By working together, we can influence decisions that affect the research process.					
11.	By working together, we can influence decisions that affect people with diabetes.					

Do you have any other comments/suggestions?

Would you like to participate in a follow-up interview about your experience?

We would like to learn more about your experience of taking part in the meeting. If you are willing to be contacted about taking part in a short follow up interview (either in person or by telephone), please provide your contact details below and a member of the research team will be in touch. If you do not wish to be contacted, please leave this section blank.

Name ___________________________________

Phone Number ____________________________

Email Address______________________________

References

1. Anderson NR, West MA. Measuring climate for work group innovation: development and validation of the team climate inventory. Journal of Organizational Behavior: The International Journal of Industrial, Occupational and Organizational Psychology and Behavior. 1998;19(3):235-58.
2. Kivimaki M, Elovainio M. A short version of the Team Climate Inventory: Development and psychometric properties. Journal of occupational and organizational psychology. 1999;72(2):241-6.
3. Song H, Chien AT, Fisher J, Martin J, Peters AS, Hacker K, et al. Development and validation of the primary care team dynamics survey. Health services research. 2015;50(3):897-921.
4. Yessis JL, Kost RG, Lee LM, Coller BS, Henderson DK. Development of a research participants' perception survey to improve clinical research. Clinical and translational science. 2012;5(6):452-60.
5. Schulz AJ, Israel BA, Lantz P. Instrument for evaluating dimensions of group dynamics within community-based participatory research partnerships. Evaluation and Program Planning. 2003;26(3):249-62.
6. Fowler Jr FJ. Survey research methods: Sage publications; 2013.
