# Supplementary material for: 'It just wasn’t going to be heard’: A mixed methods study to compare different ways of involving people with diabetes and health‐care professionals in health intervention research
Source: Health Expect. 2020 May 1;23(4):870–83. doi: 10.1111/hex.13061 (PMC7495083; doi:10.1111/hex.13061)
Supplement: Supplementary file 5 [file HEX-23-870-s005.pdf]

Supplementary File 5: Observation Guide and Grid

How is the group working overall?
**How is the group making decisions?**

**Participation / Non-participation**	**Dominance / submissiveness**	**Ingroups / Outgroups**
**Body language & Facial Expressions**	**Gaze**	**Effect of Expert / Lay knowledge**

Observations
