# Supplementary material for: 'It just wasn’t going to be heard’: A mixed methods study to compare different ways of involving people with diabetes and health‐care professionals in health intervention research
Source: Health Expect. 2020 May 1;23(4):870–83. doi: 10.1111/hex.13061 (PMC7495083; doi:10.1111/hex.13061)
Supplement: Supplementary file 6 [file HEX-23-870-s006.pdf]

Supplementary File 6: Interview Topic Guide

Interview Topic Guide

Questions	Prompts
Did you feel comfortable expressing your opinion in the group?	Why/why not?<br>Did you feel part of the group (like you belonged to the group)?
Did you feel that you could influence the decisions that the group made?	Why/why not?<br>Was your opinion listened to and considered by other group members?
Did everyone in the group have a voice in the decisions that were made?	Did certain individuals talk more at the meeting than others?<br><br>Did you feel that certain individuals had more influence over the decision-making process than others?
Do you think that the group was able to deal with conflicts that came up during the meeting?	How did the group deal with conflicts?
Did you feel pressured to go along with decisions of the group even though you might not agree?	Did you feel a sense of trust and openness between group members?
